# Identification of the minimal melanocyte-specific promoter in the melanocortin receptor 1 gene

**DOI:** 10.1186/1756-9966-27-71

**Published:** 2008-11-18

**Authors:** Stefania Miccadei, Barbara Pascucci, Mauro Picardo, Pier Giorgio Natali, Donato Civitareale

**Affiliations:** 1 Laboratory of Molecular Pathology and Ultrastructure, Regina Elena Cancer, Institute, Via delle Messi d'Oro 156, 00158 Rome, Italy; 2 Institute of Neurobiology and Molecular Medicine, CNR, Via Fosso del Cavaliere, 100, 00133 Rome, Italy; 3 Institute of Cristallography, CNR, Via Salaria, 00016 Monterotondo Stazione, Italy; 4 Laboratory of Cutaneous Physiopathology, San Gallicano Dermatological Institute, Via San Gallicano 25/a,00153 Rome, Italy

## Abstract

**Background:**

The understanding of cutaneous pigmentation biology is relevant from the biologic and clinical point of view. The binding of α-melanocortin and its specific receptor, on the plasma membrane of melanin synthesising cells, plays a crucial role in melanins biosynthesis. Furthermore, loss of *MC1R *function is associated with an increased incidence of melanoma and non-melanoma skin cancer. The expression of the α-melanocortin receptor gene is highly controlled but, at the present, region responsible for tissue-specific activity of the gene promoter has not been identified.

**Methods:**

We have cloned the genomic sequences upstream the human MC1R coding gene. A DNA fragment of 5 kilobases upstream the human MC1R encoding sequence was placed in front of a reporter gene and several deletion mutants of such fragment have been prepared. These constructs have been tested for the ability to drive the melanocyte-specific gene expression of the reporter gene using transfection experiments in melanocyte and non-melanocyte cell lines. From these experiments we identified a DNA fragment with the ability to drive the gene transcription in a tissue-specific way and we used this small DNA fragment in DNA-protein interaction assays.

**Results:**

We show that the 150 base pairs upstream the MC1R gene initiation codon are able to drive the melanocyte-specific gene transcription. Furthermore, we provide experimental evidences suggesting that on such minimal melanocyte-specific gene promoter can assemble tissue-specific complexes.

**Conclusion:**

The present results strongly imply that the transcriptional regulation of the melanocyte-specific MC1R gene requires an internal promoter located in the 150 base pairs upstream the initiation codon.

## Background

Melanocytes and neuroblasts through melanogenesis and catecholamine synthesis are capable of transforming the same precursor L-tyrosine [1]. These two pathways are potentially cytotoxic and mutagenic due to the production of reactive oxygen species [2]. A crucial role in melanins biosynthesis is played by the pro-opiomelanocortin as well as by the melanocortin receptor 1 (MC1R), the precursor of the α-melanocortin (α-MSH) and its seven-transmembrane G-protein coupled receptor, respectively [3]. The α-MSH binding to MC1R increases the level of cyclic AMP (cAMP) [4]. The adenylate cyclase pathway induces the expression of the microphtalmia-associated transcription factor (MITF), a member of the basic helix-loop-helix (bHLH) transcription factors that through binding to the E-box (CANNTG) on the gene promoters is involved in the transcriptional regulation of several melanocyte-specific genes such as tyrosinase, Tyrosinase Related Protein-1 (TRP-1) and TRP-2 [5]. Therefore, MC1R is a melanocyte differentiation marker which plays a major role in the pigmentation process in mammals. *MC1R *variants are also associated with increased risk of malignant melanoma in a variety of populations [6,7], MC1R gene product has been proposed as a new marker and a putative therapeutic target for uveal melanoma [8]. Although fair skin is a well-known risk factor for skin cancer, most of the aforementioned studies conclude that increased skin cancer susceptibility due to *MC1R *variation cannot be explained solely on the basis of pigmentary phenotype thus making the effects of the MC1R variants on skin cancer risk unrelated [9].

Although MC1R has been detected in different cell lineages, it is mainly expressed in melanocytes. Its gene expression is regulated at transcriptional, post-transcriptional and translational levels by its agonist, α-MSH, and the antagonists, agouti signal protein and agouti related protein [10].

The MC1R gene promoter has been identified and the minimal region exhibiting promoter activity has been located between nucleotides -517 and -282 from the initiation of translation [11]. Experimental evidences revealed that the gene promoter includes several E boxes able to bind both MITF and Upstream Stimulating Factor-1 [11,12]. It has been shown that MITF activates the MC1R gene promoter in cultured mast cells and in co-transfection experiments in NIH3T3 cells [13,14], thus raising the question about the melanocyte-specific expression of MC1R. In this regard, Moro et al. have shown that the minimal MC1R gene promoter displays the same activity in receptor positive as well as negative cells [11]. This suggests that the cis-acting region responsible for tissue-specific expression of the MC1R has not been jet identified.

In the present study we have addressed the identification of the sequences, in the human MC1R gene promote, which are involved in the melanocyte-specific expression. We demonstrate that the minimal promoter with melanocyte-specific activity resides in the fragment of 150 base pairs upstream of the start codon.

## Methods

### Genomic screening, cloning and deletion mutants

In order to identify the cosmid containing the MC1R gene a pool of cosmids clones, containing a human genomic library, were screened by PCR using the oligos 5'-atggctgtgcagggatcccagag-3' and 5'-gtgcaccggcctccagcaggaggatg-3'. The library was obtained from the YAC Screening Center (Milan, Italy). The selected cosmid was digested with Mlu1 and BamH1 and the 5 kb fragment was cloned in pGL3 Basic (Promega Madison WI USA) digested with Mlu1 and Nco1. The BamH1 and the Nco1 sites were blunted by fill-in reactions in presence of deoxinucleotides and the large Klenow fragment. The new construct, pFull, was confirmed by restriction analysis and by direct sequencing of the insert borders. pFull-A was generated digesting pFull with Aat2 and self-ligation of the large fragment. pFull-Bs derives from the self-ligation of the large fragment generated by the pFull digestion with BssH2. The construct pFull-MBs has been generated by the self-ligation of the vector pFull digested with Mlu1 and BssH2, both fragment ends were blunted by fill-in reactions in presence of deoxinucleotides and the Klenow enzyme. To generate pFull-Bg, the DNA fragment of about 1.8 kb obtained from pGL3 Basic digested with Bgl2 and Xba1 was cloned in the vector obtained digesting pFull-MBs with the same enzymes. pFull-MBg derives from the self-ligation of the large fragment of pFull-MBs digested with Bgl2 and Mlu1, both fragment ends were blunted by fill-in reactions in presence of deoxinucleotides and the large Klenow fragment. All the deletion mutants were confirmed by restriction analysis or by direct sequencing.

### Cell lines and transfection experiments

The HeLa and NIH 3T3 cell lines, (kindly provided by Dr. M. Alimandi) as well as the MeWo cell line were cultivated in D-MEM (Invitrogen Life Technologies, Milan, Italy) supplemented with 10% of foetal calf serum, 10 IU/ml of penicillin and 10 μg/ml streptomycin at 37°C in humidified atmosphere of 5% CO_2_-95% air. The transfection experiments were performed in 6 well vessels by Lipofectamine 2000 reagent Invitrogen following the manufacturer instructions. The luciferase reporter vector used at 0.4 μg and 50 ng of the pCMV-β-galactosidase plasmid were used to determine transfections efficiency. 48 hours after transfections the cell extracts were prepared using the Report Lysis Buffer (Promega, Milan Italy). The luciferase and β-galactosidase assays were performed as previously reported [15]. Transfection experiments were done in triplicate and performed at least three times. For each experiment the mean of three independent experiments is reported. The statistical analysis, performed utilising the raw data, of all transfection experiments resulted in *P *< 0.05. For each experiment, the t-test (the probability associated with the t-test) was calculated versus the respective control group.

### Electrophoretic mobility shift assay (Band-shift) and run-on experiments

The band-shift assays were performed using nuclear proteins extracted from MeWo and from NIH3T3 cells. The nuclear proteins were extracted according to Shapiro et al. [16]. The DNA probe was prepared by PCR using the pFull vector as template and the oligonucleotides 5'-agatctgggggtgcccagatggaagg-3' and 5'-agtcctgtccaggaagcaggaaggag-3'. The forward oligonucleotide was labelled with γ-ATP ^32^P and T4 Polynuclotide kinase. The PCR amplicon was run on 2% agarose gel, purified and electroeluted. The DNA binding assays were assembled on ice in buffer D (20 nM Tris/HCl pH 7.9, 80 mM NaCl, 0.1 mM EDTA, 1 mM DTT, 0.5 mM PMSF, 10% glycerol) with 4 μg polyd(I-C) and with10 μg of the extracted proteins. The reaction was started adding the ^32^P labelled DNA fragment. After 20 minutes at room temperature the reaction was loaded onto a 4.5% polyacrylamide gel in 0.5× Tris/Borate/EDTA buffer and elettrophoresed at 200 V for 4 hours at 4°C.

Preparation of nuclei and nuclear run-on assays were done essentially as previously described [17]. Briefly, nuclei were harvested from approximately 500000 cells by gentle vortexing in 700 μl of ice-cold lysis buffer (10 mM NaCl, 3 mM MgCl_2_, 10 mM Tris-HCl at pH 7.4, 5 mM dithiothreitol, 0.5% Nonidet P-40). Nuclei were pelleted by centrifugation, and cytoplasmic RNA was isolated from the supernatant by adding 2 volumes of guanidinium thiocyanate buffer. The nuclei were resuspended in an equal volume of storage buffer (40% glycerol, 50 mM Tris-HCl at pH 8.5 mM MgCl_2_, 0.1 mM EDTA) and frozen at -80°C until assayed. To label nascent transcripts, nuclei were thawed on ice and incubated in 25 mM HEPES (pH 7.4), 2.5 mM MgCl_2_, 2.5 mM dithiothreitol, 75 mM KCl, and 5% glycerol in the presence of 0.35 mM each ATP, GTP, CTP, and 0.4 μM UTP plus 200 μCi of α-UTP ^32^P (3000 Ci/mM, DuPont NEN, Boston MA USA) for 30 min at 30°C. The reaction was stopped by digestion with RNase-free DNase I for 15 min at 37°C followed by digestion with proteinase K in the presence of 0.1% SDS and extraction with phenol/chloroform. Nucleic acids were precipitated with cold 10% trichloroacetic acid, sequentially washed with 5% trichloroacetic acid and ethanol, and resuspended in 10 mM Tris-HCl (pH 7.4), 1 mM EDTA, and 0.5% SDS. Hybridization of run-on transcription products was performed in hybridization buffer (0.5 M phosphate buffer at pH 7.2, 3.5% SDS, 33% formamide, 1 mg/ml bovine serum albumin, 1 mM EDTA, 20 μg/ml yeast tRNA) at 55°C overnight. Blots were washed to 0.2 × SET (1 × SET is 150 mM NaCl, 30 mM Tris-HCl, pH 7.8, 2 mM EDTA), 0.1% SDS at 55°C, and exposed in a PhosphorImager cassette. The membrane was scanned and analyzed using the PhosphorImager system with the ImageQuant software (Molecular Dynamics from Amersham Biosciences Europe GmbH)

## Results

### MC1R promoter activity in melanocyte and non-melanocyte cell lines

**Figure 1:**
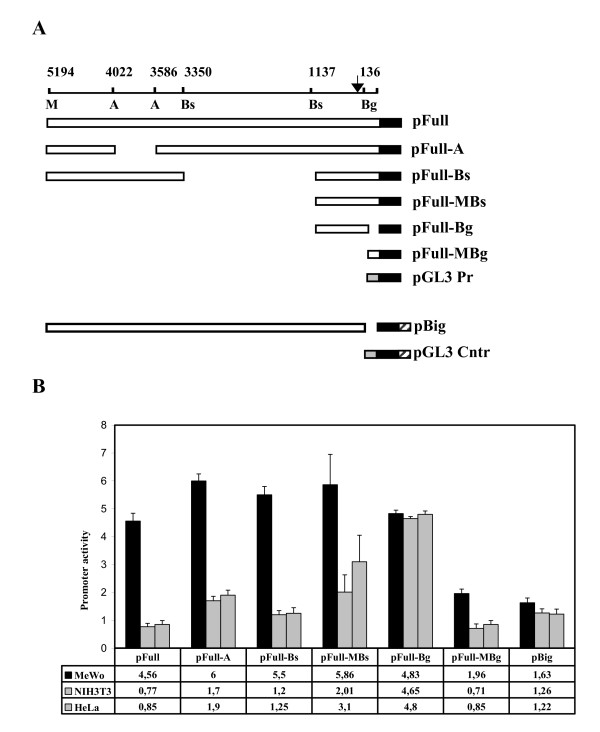
**Transfection experiments.** Schematic representation of MC1R-Luciferase reporter constructs and corresponding activities after transfection into MeWo, NIH3T3 and HeLa cell lines. A. We schematically illustrate, as empty box, the MC1R promoters driving the luciferase gene shown as a black box. The arrow schematically represents the major start of transcription as we have determined it by primer extension experiments both in MeWo and in NIH3T3 cells transfected with the pFull vector (data not shown). It is worth to mention that this result is in agreement with the start of transcription of the human MC1R gene previously reported [4], [5]. M, A, Bs and Bg indicate the restriction enzymes Mlu1, Aat2, BssH2 and Bgl2, respectively. B. pFull and the described deletion mutants have been transfected in MeWo, NIH 3T3 and HeLa cell lines. The promoter activity in MeWo (black), NIH 3T3 (gray) and Hela (stripe) are reported as percentage of the SV40 promoter of pGL3 Promoter vector arbitrarily considered as 100. The pBig promoter activity has been compared with the pGL3 Control activity, and in this case the transcriptional activity of pGL3 Control was arbitrarily considered, in all cell lines, as 100.

**Figure 2:**
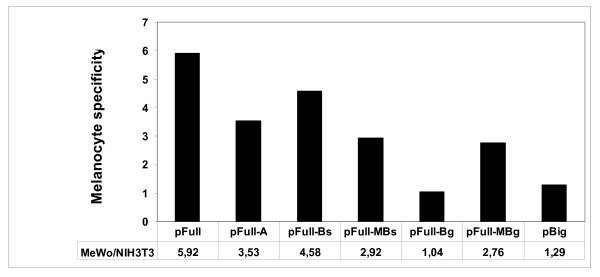
**Melanocyte-specificity**. Using the results shown in figure 1 we report an index of melanocyte-specificy. It has been obtained dividing the mean promoter activity obtained in MeWo by the mean promoter activity measured in NIH 3T3.

**Figure 3:**
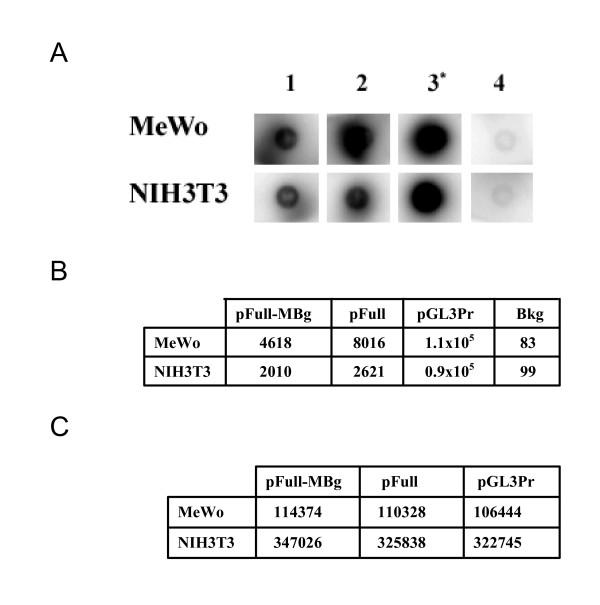
**Run-on experiment**. A. Dot blot. 1, 2 and 3 indicate the transfections with pFull-MBg, pFull and pGL3Promoter, respectively. In the experiments shown in lanes 1, 2 and 3, the nylon membrane has been spotted with 2 μg of pGL3Basic and in the experiment shown in lane 4, it has been spotted 2 μg of pBluescript to measure the background of the hybridization with the labelled nuclei of the cell lines transfected with pGL3 Promoter. * indicates that the exposure time is of about 2 hours whereas in 1, 2 and 4 the blots have been exposed over-night. B. Each dot blot has been counted in the β-counter and the cpm are reported. Bkg stays for background and indicates the cpm of the experiments shown in lane 4. C. As transfection normaliser we have co-transfected, in all the experiments, 200 ng of pCMV-Seap2 vector. The Seap activity was measured with SEAP Reporter Gene Assay (Roche). Since the transfection efficiency in NIH3T3 is about three times higher than in MeWo cells (see the Seap2 activity in section C), we have used, in the run-on experiments, one third of the purified NIH3T3 nuclei and all the nuclei purified from MeWo.

With the aim of identifying the minimal tissue-specific promoter involved in the melanocyte-specific MC1R gene transcription, we have transiently transfected the vector pFull, containing the 5 kb fragment upstream the MC1R encoding sequence, or the vectors containing the deletion mutants shown in figure 1A. The experiments were performed in human melanoma MeWo cell line, expressing the MC1R, and in NIH 3T3 cells, a murine cell line of fibroblasts not expressing the receptor. As further negative cell line we have used the HeLa cell line. The results of the transfection experiments are reported in figure 1B. It shows that the construct with 5 kb of the genomic sequence upstream of the MC1R gene, pFull, drives the expression of the reporter gene much more efficiently in melanocytes than in non-melanocyte cells. Similarly, the constructs, pFull-A, pFull-Bs, pFullMBs and pFull-MBg show a higher transcriptional rate in MeWo cells. At variance, the promoter in pFull-Bg vector shows, in both cell lines, the same activity. The promoters in pFull-MBs and pFull-Bg differ only for the 150 bp Bg. That are absent in pFull-Bg. Consistently, the pFull-MBg vector containing only the first 150 bp, although with weaker activity respect to the promoter of pFull, is still tissue-specific. Hence, this result suggests that the 150 bp upstream the coding gene plays a crucial role in the melanocyte-specific regulation of the MC1R gene promoter. In agreement with this result we show that the promoter in pBig has about the same activity in all three cell lines, figure 1B. The large fragment of about 5 Kb but deleted of the first 150 bp, present in pBig, drives the reporter gene transcription at similar levels in NIH 3T3, Hela and in MeWo cell lines. As previously described in "Materials and Methods", this large fragment, -5 Kb/-150 bp, has been cloned in pGL3 Enhancer (Promega), thus the luciferase gene is under the transcriptional control of the SV40 enhancer, present in the vector, and of the inserted DNA fragment. Therefore, as control of pBig we have used pGL3 Control, where the transcriptional activity of the luciferase gene is under the regulation of both the SV40 Enhancer and SV40 Promoter. pBig and pGL3 Control, show a comparable activity in the MC1R positive and negative cell lines indicating, once again, that the initial 150 bp are required for melanocyte-specific transcription of the MC1R gene, figure 1B. Moreover, we show the melanocyte specificity of the previously described promoters, Figure 2. For each promoter the mean activity obtained in melanocytes was divided by the mean activity obtained in NIH 3T3 fibroblasts. Therefore, the MeWo/NIH value represents an index of the melanocyte-specificity. Both constructs with the promoters lacking the first 150 bp, pFull-Bg and pBig, show a value close to 1 indicating that their activity was similar in the two cell lines. Therefore, the results shown in figure 1 and 2 strongly suggest that the 150 bp upstream of the MC1R encoding gene are necessary for the melanocyte-specific activity of the gene promoter. In order to demonstrate that the preferential expression of pFull and of pFull-MBg in melanocytes is regulated at transcriptional level, we have performed run-on experiments in MeWo and NIH3T3 transfected cells, figure 3. We have transiently transfected the two cell lines with pFull or with pFull-MBg or with pGL3 Promoter and to normalize for transfection efficiency with pCMV-Seap2 vector as well. After 48 hours, we have measured the Seap activity in the medium and we have purified the nuclei from the cells. In figure 3A we show the run-on experiment. In figure 3C we report the values of the Seap assays and in figure 2B we show the run-on results. Since we have performed the run-on experiments taking into account the transfection efficiency, the results shown in figure 3B, directly indicates that the reporter gene in pFull and in pFull-MBg is better transcribed in MeWo rather than in NIH3T3 cells. Therefore, the differential activity of the MC1R gene promoter, both in pFull and in pFull-MBg, shown in figure 1 with transient transfection experiments is regulated at transcriptional level.

### Melanocyte-specific DNA-protein complex

**Figure 4:**
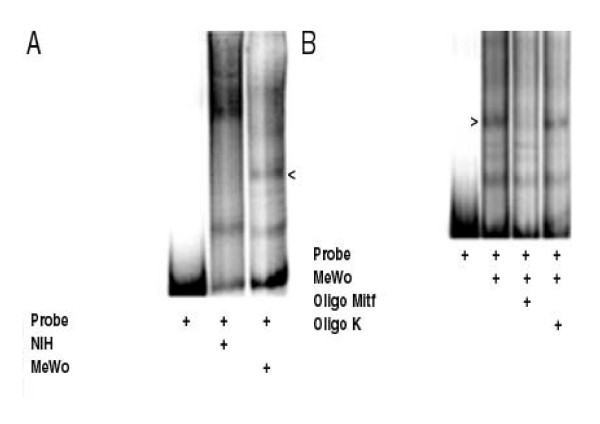
**Band-shift assay**. Characterization of the complexes assembled on the minimal melanocyte-specific MC1R gene promoter. The labeled DNA fragment was incubated with the nuclear proteins extracted from NIH3T3 or MeWo as indicated. The arrow indicates the most evident tissue-specific complex.

To support the results obtained in transfection experiments we have performed band-shift experiments using as probe the 150 bp upstream the human MC1R coding gene, the entire minimal promoter identified in the transfection experiments shown in figure 1. Such a probe was assembled, in a band-shift assay, with the nuclear proteins extracted from NIH 3T3 cells or from MeWo cells. In figure 4A we show that, together with some similar complexes, there are some complexes formed only with the nuclear proteins from melanocytes. The most evident of the melanocyte-specific complexes is indicated by the arrowhead. Since MITF is a crucial element of the melanocyte-specific gene transcription and since it has been shown that it activates the MC1R gene promoter we have envisaged that MITF could be present in the melanocyte-specific complex shown in figure 4A[18]. In figure 4B we show that the melanocyte-specific complex assembled on the minimal MC1R tissue-specific promoter is specifically competed by the presence, in the assay, of the double strand oligonucleotide containing the MITF binding sequence derived from the tyrosinase promoter, on the contrary the same amount of an unrelated oligonucleotide, k oligo, does not affect the complex. Therefore, this result suggests that MIFT is present in the complex assembled on the 150 bp DNA fragment containing, as shown in figure 1, the minimal melanocyte-specific MC1R gene promoter. Hence, with the band-shift assay, we have identified a melanocyte-specific complex assembled on the same DNA fragment that in transfection experiments shows a melanocyte-specific promoter activity.

## Discussion

In this study we have identified in the 150 bp, upstream the ATG codon of the MC1R gene, the cis signal conferring to the minimal gene promoter the melanocyte-specific gene transcription. The characterization of the 5'-flanking region of the human MC1R has been performed by Moro et al. [11], they have analyzed about 3 kb of the human genomic sequences upstream the MC1R start codon, from -282 to -3200, and they have identified the minimal promoter region between the nucleotides -517 and -282. However, the activity of all the analyzed constructs showed similar promoter activity in both MC1R expressing and non expressing cells. Therefore, we propose to modify such a MCR1 gene promoter definition including the 150 bp upstream the initiation codon. The human MC1R gene shows multiple transcription initiation sites dispersed over a region of about 600 nucleotides spanning from -344 to -936 [10,11]. Therefore, our results identify the melanocyte-specific cis promoter region in the DNA sequence for the 5' untranslated region [5'-UTR] of the messenger RNA. The formation of tissue-specific DNA-protein complexes down-stream of the initiation of transcription has been already described. Besides the internal promoters of the RNA polymerase III transcribed genes and from complexes assembled on the enhancer sequences that are often located at the 3' of the gene, several RNA Polymerase II transcribed genes have cis elements located in the genomic region transcribed in the 5'-UTR and described as internal promoter. The transcription of macrophage IGF-I exon 1 is positively regulated by the 5'-UTR region [19] and, similarly, the CD28 gene is transcriptionally regulated by cis sequences located in the first exon upstream of the initiation codon [20].

On the basis of the results of the transfection data a further consideration can be made. The melanocyte-specific expression of the MC1R gene might be achieved not only by the positive activity of tissue-specific transcription factors, such as MITF [12], expressed in the pigmented cells but also through DNA-protein complexes able to repress transcription in non-melanocytes. In fact, in the transfection experiments in NIH 3T3 cells, the most significant difference in promoters strength is the increased activity of pFull-Bg. Therefore, the possibility that a repressing complex has been removed by the deletion of the first 150 bp in pFull-Bg should be considered. Therefore, it is possible to envisage that in non-melanocyte cells a potential repressor could bind in the first 150 bp. In this regard, it is worth to note that the band-shift experiment shows several NIH3T3 specific complexes as well. This strategy to regulate tissue-specific gene expression would parallel the regulation exerted by REST/NRSF in neuronal cells. The main function of REST/NRSF in fact is to repress expression of some neuronal genes in non-neuronal cell types and in neuronal progenitor cells [21].

## Conclusion

Although the present findings obtained with transient transfection experiments, need further analysis of the transcription factors assembled on the MC1R gene promoter, collectively they demonstrate that the transcriptional regulation of the melanocyte-specific MC1R gene requires an internal promoter located in the 150 bp upstream the initiation codon.

## Competing interests

The authors declare that they have no competing interests.

## Authors' contributions

DC, PGN and MP conceived of the study, participated in its design, discussed the results and helped to draft the manuscript. SM carried out the transfection and the run-on experiments. BP carried out the vectors construction and the band-shift assays. DC wrote the manuscript. All authors read and approved the final manuscript.
